# Access to health care for people with disabilities in rural Malawi: what are the barriers?

**DOI:** 10.1186/s12889-020-08691-9

**Published:** 2020-06-01

**Authors:** Josephine A. K. Harrison, Rachael Thomson, Hastings T. Banda, Grace B. Mbera, Stefanie Gregorius, Berthe Stenberg, Tim Marshall

**Affiliations:** 1 Bristol Medical School, University of Bristol, Bristol, UK; 2 Collaboration for Applied Health Research & Delivery, Liverpool School of Tropical Medicine, Liverpool, UK; 3 Research for Equity And Community Health (REACH) Trust, Lilongwe, Malawi; 4 LHL International, Oslo, Norway; 5 School for Policy Studies, University of Bristol, Bristol, UK

**Keywords:** Health care access, Barriers, Disability, Impairment, Malawi, Rural, In-depth interviews

## Abstract

**Background:**

People with disabilities experience significant health inequalities. In Malawi, where most individuals live in low-income rural settings, many of these inequalities are exacerbated by restricted access to health care services. This qualitative study explores the barriers to health care access experienced by individuals with a mobility or sensory impairment, or both, living in rural villages in Dowa district, central Malawi. In addition, the impact of a chronic lung condition, alongside a mobility or sensory impairment, on health care accessibility is explored.

**Methods:**

Using data from survey responses obtained through the Research for Equity And Community Health (REACH) Trust’s randomised control trial in Malawi, 12 adult participants, with scores of either 3 or 4 in the Washington Group Short Set (WGSS) questions, were recruited. The WGSS questions concern a person’s ability in core functional domains (including seeing, hearing and moving), and a score of 3 indicates ‘a lot of difficulty’ whilst 4 means ‘cannot do at all’. People with cognitive impairments were not included in this study. All who were selected for the study participated in an individual in-depth interview and full recordings of these were then transcribed and translated.

**Results:**

Through thematic analysis of the transcripts, three main barriers to timely and adequate health care were identified: 1) Cost of transport, drugs and services, 2) Insufficient health care resources, and 3) Dependence on others. Attitudinal factors were explored and, whilst unfavourable health seeking behaviour was found to act as an access barrier for some participants, community and health care workers’ attitudes towards disability were not reported to influence health care accessibility in this study.

**Conclusions:**

This study finds that health care access for people with disabilities in rural Malawi is hindered by closely interconnected financial, practical and social barriers. There is a clear requirement for policy makers to consider the challenges identified here, and in similar studies, and to address them through improved social security systems and health system infrastructure, including outreach services, in a drive for equitable health care access and provision.

## Background

Equitable access to timely and adequate health care is an intrinsic component of overall equity in health and lack of it may be both an indicator and “contributory cause” [1] of a population’s health inequalities. An array of diverse barriers, occurring at various points along the pathways to care, restricts health care accessibility for many individuals and groups globally. Especially disproportionate though, is the extent and impact of the barriers encountered by the world’s largest minority group: people with disabilities [2].

It is estimated that over one billion of the global population are disabled in some way and, with this figure on the rise, the World Health Organisation (WHO) has declared a need for more qualitative research in order to better understand the “lived experiences of people with disabilities” [2]. A recent systematic review [3] highlights an especially “urgent” requirement for research in low- and middle-income countries as it finds strong evidence for both cause and consequence associations between disability and poverty.

Malawi is a low-income country which has a health system that is significantly limited in its number of facilities, provision of drugs, medical personnel and amount of funding [4]. Given that 85% of the country’s population live in rural communities [5], the impact of this on the accessibility of health care, for many, is further restricted by lack of physical infrastructure and financial means. Among those living rurally, people with disabilities are especially vulnerable to health care access barriers [6].

### Health care accessibility

The WHO describes ‘accessibility’ - encompassing physical, economic and information accessibility as well as non-discrimination - as one of the four elements of the right to health, along with availability, acceptability and quality (the AAAQ framework) [7].

The ‘Three Delays model’ [8], first put forward by Thaddeus and Maine in the context of maternal mortality yet applicable to all aspects of health, identifies three critical points of potential delay in accessing of health care: “(1) delay the decision to seek care; (2) delay arrival at a health facility; and (3) delay the provision of adequate care” [8]. This model may be generalised to other contexts, with the extent of each delay often varying for different communities and individuals [8]. Importantly, the interpretation of the model in this paper is such that the accountability of each delay is with the system and society, not the person with the disability.

In a more recent paper exploring factors involved in reaching the health system interface, Levesque et al. [9] build upon the Three Delays model and define health care accessibility as the sum of five dimensions: “Approachability; Acceptability; Availability and accommodation; Affordability; Appropriateness” [9]. In explanation of these, Levesque et al. present five accompanying abilities of the health service user: “Ability to perceive; Ability to seek; Ability to reach; Ability to pay; Ability to engage” [9]. Their analysis, which includes an overview of various categorisations of the factors known to influence a person’s access to suitable and timely health care, highlights the complex nature of health care accessibility and, in turn, its barriers.

It is important to consider the contextual variation of health care accessibility by exploring how the weight of certain dimensions and abilities may shift with respect to specific regions, societal contexts and health systems.

**Fig. 1:**
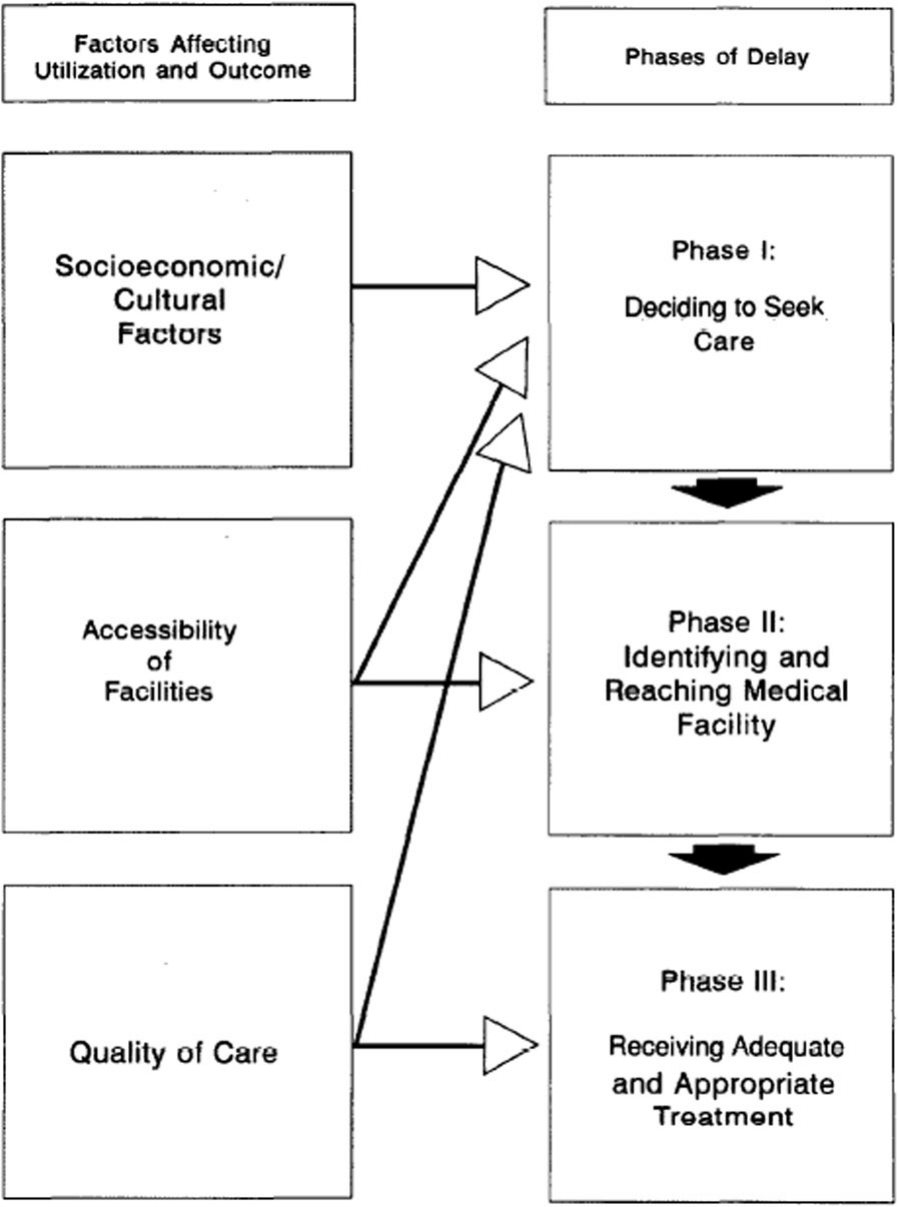
Thaddeus and Maine’s Three Delays Model [8]. Licence number for reproduction of figure: 4797000461627

**Fig. 2:**
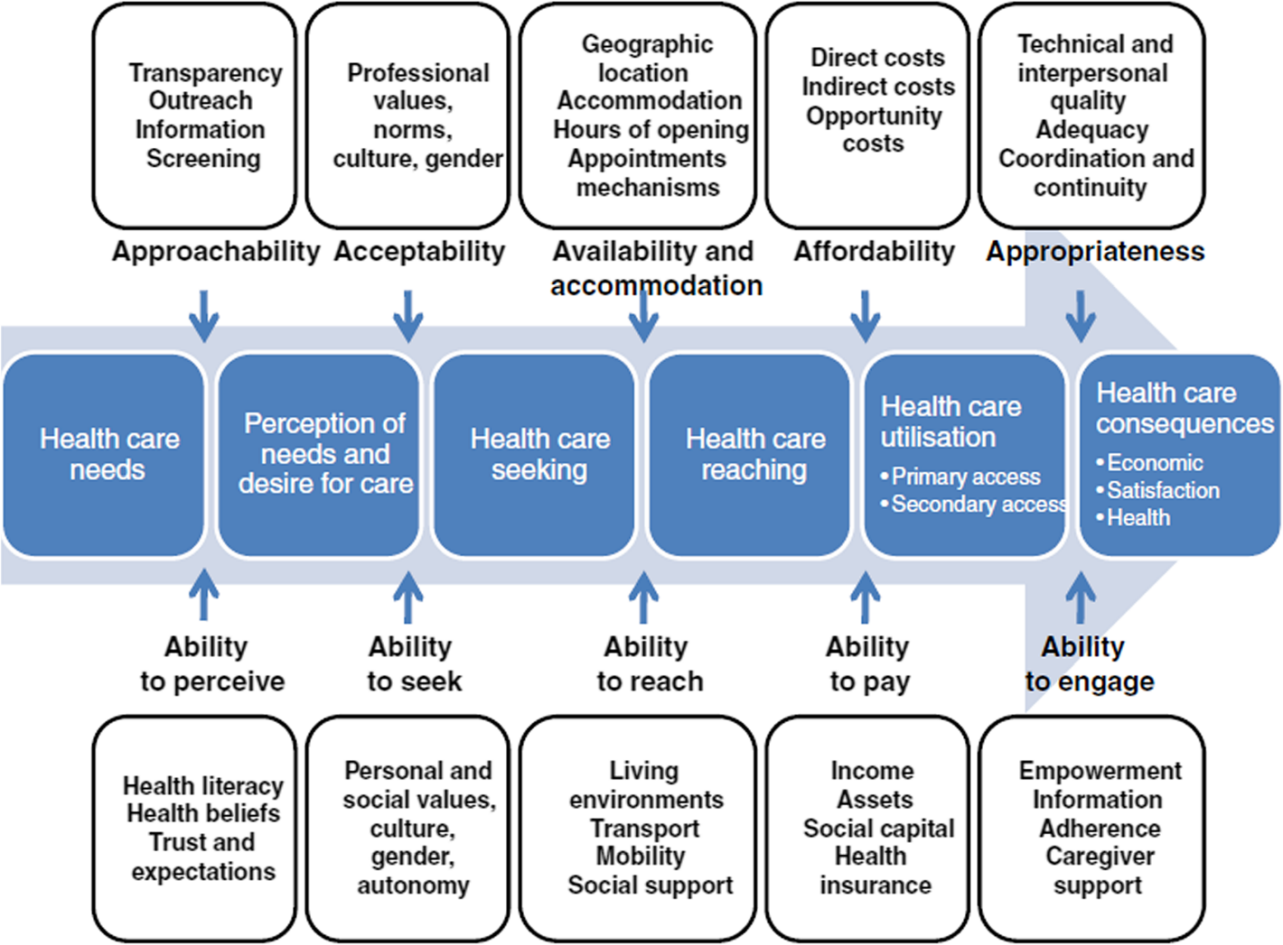
Levesque et al.’s Conceptual Framework of Access to Health Care [9]. The reproduction of this figure is permitted under the terms of the Creative Commons Attribution License (http://creativecommons.org/licenses/by/2.0), which allows unrestricted use, distribution, and reproduction in any medium, provided the original work is properly cited.

This paper will use correlations with Thaddeus and Maine’s Three Delays and Levesque et al.’s accessibility model, shown in Figures 1 and 2, as tools to support explanations of the financial, practical and social barriers faced by our participants in rural Malawi.

### Health care accessibility for people with disabilities in Malawi

Findings from a large-scale household survey presented by Eide et al. reveal that, like disability itself, barriers to health care access for people with disabilities are diverse. In Malawi and three other African countries, four main access barriers are identified: “lack of transport, availability of services, inadequate drugs or equipment, and costs” [10] . These findings broadly align with the accessibility dimensions, ‘availability and accommodation’ (for the first two findings), ‘appropriateness’ and ‘affordability’ respectively. This suggests that health care accessibility for people with disabilities in Malawi is more likely to be restricted by the abilities to reach, pay for and engage with the services available than to perceive the need for and to seek health care.

In contrast, a study by Grut et al. [11], finds “lack of information and knowledge” to be a barrier to tuberculosis services for people with disabilities in Malawi. Discussing the role that understanding disease plays in accessing timely and adequate health care, Grut et al. imply that the ‘ability to perceive’ the need for care is a limiting factor of accessibility in this setting.

There are disagreements in the literature about the role of community and health care workers’ attitudes towards disability in providing a barrier to health care access. Most authors, including Eide et al. [10], conclude that attitudinal barriers exist. Meanwhile, Grut et al. [11], who explore the role of health care workers’ attitudes but not those of community members, find that people with disabilities are treated respectfully by medical personnel.

### Research objectives

A review of the literature on the health inequities experienced by people with disabilities presents a clear problem: people with disabilities face restricted access to health care. Given this, identification of the barriers to health care for the world’s largest minority group remains a “leading research priority” [12]. There has been a lack of in-depth exploration of the barriers to general health care access experienced by people with mobility or sensory impairments in rural low-income settings. It is this gap in the literature which the present study, conducted in Dowa district, central Malawi, is designed to address.

The findings from this qualitative study are being used to complement the data obtained in the Triage II Plus project (Trial Registration Number: NCT02127983) [13] in which it is nested. Triage II Plus, which is being carried out by the Research for Equity And Community Health (REACH) Trust in Malawi, is a trial investigating the approaches to improve detection and management of chronic airways disease. For this reason, our study will also examine whether the access barriers differ for people who have a chronic lung condition alongside their mobility or sensory impairment.

The aim of this nested study is to explore the barriers to timely and adequate health care experienced by people with mobility or sensory impairments, or both, in rural Malawi and there are two objectives within this:

1. To identify the main access barriers faced by people with disabilities and explore the impact of these on their pathway to care.
2. To understand how the barriers faced by people with disabilities might differ from those faced by the wider population, with reference to findings in the surrounding literature.

It is important to explain at this point what we mean by the terms ‘impairment’ and ‘disability’, both of which are used throughout the paper. In an exploration of the two terms with reference to the social model of disability, Goering [14] highlights the definition of impairment as a functional limitation, and defines disability as “a disadvantage that stems from a lack of fit between a body and its social environment”. In this paper, we use ‘impairment’ when referring to the participants’ characteristics because our participants identified themselves as having impairments. Meanwhile, in order to fit with current discourse in the field, we use the term ‘disability’ when speaking more broadly about the barriers and their implications because this is where we find that societal factors are causing the impairment to become a disability.

## Methods

### Recruitment

The fieldwork for this study was carried out in April 2018 alongside the Triage II Plus Endline Survey [13] which provided the data for selection and recruitment of participants. In order to coordinate with local research assistants, and because the data collection for the current study was conducted at the same time as that of the Triage II Plus project, the first 12 survey respondents who were considered to meet our inclusion criteria and could be located using the household GPS coordinates provided, were recruited. The inclusion criteria required participants to be over 18 years of age and to have a mobility impairment, sensory impairment, or both, as identified through the WHO Washington Group Short Set (WGSS) Questions [15] within the survey. The respondents who scored 3 (‘a lot of difficulty’) or 4 (‘cannot do at all’) for the questions concerning their ability to see, hear or walk were included in our study. Table 1 shows the total numbers of scores ≥3 and scores < 3 recorded in the Triage II Plus Endline Survey for each of these WGSS questions.

**Table 1:** Triage II Plus Endline Survey figures

	PAL^a^	PAL+^b^	Control	TOTALS:
Number interviewed	4447	4145	4365	**12,957**
Difficulty seeing ≥3	13 (0.3%)^c^	16 (0.4%)	16 (0.4%)	**45 (0.3%)**
Difficulty seeing < 3	4434 (99.7%)	4129 (99.6%)	4349 (99.6%)	**12,912 (99.7%)**
Difficulty hearing ≥3	24 (0.5%)	10 (0.2%)	17 (0.4%)	**51 (0.4%)**
Difficulty hearing < 3	4423 (99.5%)	4135 (99.8%)	4348 (99.6%)	**12,906 (99.6%)**
Difficulty walking ≥3	54 (1.2%)	47 (1.1%)	28 (0.6%)	**129 (1.0%)**
Difficulty walking < 3	4393 (98.8%)	4098 (98.9%)	4337 (99.4%)	**12,828 (99.0%)**

A table showing the total number of people with scores ≥3 and number with scores < 3 in each of the WGSS questions asked within the Triage II Plus Endline Survey

^a^PAL = Practical Approach to Lung health intervention

^b^PAL+ = PAL plus community outreach intervention

^c^Figures in brackets refer to the percentage of the total in that category

Due to limitations in time, specialist experience and expertise, people with a cognitive impairment or mental illness, who were identified by the Triage II Plus interviewers using the information in respondents’ health passports, were excluded from our current study. The recruitment of the participants depended on our ability to physically meet with eligible Triage II Plus respondents at their households, for which we had the GPS coordinates. There were two respondents whom we were unable to locate at home, despite two attempts, hence these people were not recruited, and the next two eligible respondents were recruited instead. This means that we had to attempt to locate a total of 14 Triage II Plus respondents in order to recruit 12. There were no people approached who refused to take part or who withdrew their consent having initially agree to participate. The 12 people recruited lived across 11 different rural villages in the broad catchment area of Chinkwiri health centre in Dowa district, central Malawi. Participation in the study was voluntary and all people interviewed gave informed consent beforehand with a signature or stamp mark on the consent form. Participants with limited literacy and those with visual impairments were given a verbal explanation of the information on the form before giving their consent with a stamp mark.

The Triage II Plus survey responses were also used to gather data about participants’ baseline characteristics, specifically age, gender and type of impairment. This information, along with self-reported presence or absence of a chronic lung condition, allowed for comparisons between the barriers experienced by people of similar or differing profiles in the analysis phase of the study. Recruitment of the 12 earliest locatable adult respondents with mobility impairments, sensory impairments, or both, fortunately provided a sample with considerable variation in participant profiles, as shown in Table 2.

**Table 2:** Participants per category (*n* = 12)

Characteristic	Men	Women	N
Participants aged >18 ≤29 years:	2	3	5
Participants aged ≥30 years:	2	5	7
Participants with a mobility impairment^a^:	3	1	4
Participants with a sensory impairment^a^:	1	1	2
Participants with a mobility and sensory impairment^b^:	0	2	2
Participants with mobility impairment and a chronic lung condition^c^:	2	1	3
Participants with a sensory impairment and a chronic lung condition^c^:	1	0	1

A table showing the number of participants, split by gender, in each category for age, and type of impairment(s) and presence or absence of a chronic lung condition

^a^These participants identified as having a single impairment

^b^These participants identified as having a dual impairment

^c^These participants identified as having an impairment and a lung condition (which may or may not be related to each other)

### Data collection

This study has been designed to obtain high-quality and detailed accounts about participants’ experiences as people with disabilities living in rural Malawi, regarding their navigation of pathways to care and perceptions of barriers to health care access. In order to achieve this, the interviews used aspects of the ‘McGill Illness Narrative Interview’ [16] structure.

The choice of questions and accompanying prompts in the topic guide (Additional File 1), which was developed specifically for the purposes of this study, was partially informed by Thaddeus and Maine’s Three Delays model [8] and the accessibility dimensions and abilities described by Levesque et al. [9]. In order to minimise restrictive interview structure and maximise depth of discussion and insight, the open questions were simply used to guide the illness narrative described by the participant [17].

The semi-structured interviews were carried out by the principal researcher (JH) and an experienced interpreter, who translated between English and Chichewa. All interviews took place in the participants’ own homes and the people present were the participant, JH (female), the research team interpreter (female), and usually one of the participant’s family members. The full interviews, which each lasted approximately one hour, were recorded using a digital voice recorder.

In this paper, the interviewees have been referred to using pseudonyms. The first time each participant is mentioned or quoted, the pseudonym is followed by their age, gender, and the types of impairments which they identified themselves as having. After this, just the pseudonym and age are given. Three of the four participants with a hearing impairment used a combination of lip-reading and interpretation by a family member to understand the questions and were usually able to respond autonomously. When communication with the interviewer became difficult, however, the family member responded on the participant’s behalf.

There were also two cases where the entire interview was carried out via a family member interpreter. One was a participant with a hearing impairment, Nathaniel, whose mother was his interpreter. The other was a woman, Esther, whose grandmother acted as her interpreter because the condition causing Esther’s mobility impairment also rendered her unable to speak. It has been made clear in the findings when a quote is directly from a participant and when it is from a participant’s family member interpreter. It is acknowledged that interview answers obtained in this way, particularly when the interpreters are family members, are less reliable than those given directly by the respondent and this is recognised as a limitation of the study. In light of this, none of the conclusions drawn are based solely on findings from comments made by family member interpreters, rather they are each primarily supported by responses directly from the participants.

In a similar way, the language barrier and use of a translator might be considered a limitation. In this study, however, our translator was trained to lead each section of the interview and to translate back to the principal research periodically. The principal researcher could then request further exploration of certain responses and advise the translator on how to guide the next part of the interview. Research shows that allowing a local translator to lead the interview is of great benefit to the quality of the information obtained [18]. This is because conducting the interview in the participant’s own language not only allows the interview data to be captured in its natural form, but also allows a strong rapport to be built with the participant, who generally feels more relaxed speaking to someone of the same nationality and tongue [18]. The literature on the impact of translations in qualitative research also favours the approach used in the current study, which is to transcribe the interview recordings then translate the transcripts into English [19, 20].

### Analysis

After carrying out the 12 interviews, the full audio recordings were transcribed verbatim and translated into English by the same interpreter who had assisted with the interviews. Thorough thematic framework analysis was carried out by manually coding the interview scripts to identify common as well as divergent experiences and opinions. The open coding approach adopted allowed for all new and perhaps unexpected themes to be identified as well as those anticipated in advance based on the literature review [21]. NVivo qualitative data analysis software was an additional tool used to manage the data, and to label and explore themes [22].

## Results

The findings of this study are presented in five sections: the first three sections are dedicated to the three key findings (each of which is subdivided into three components), the fourth section, ‘Attitudinal Barriers’, presents the findings about two kinds of potential attitudinal barriers, and the fifth section is on the identification of ‘time’ as a common underlying factor.

### Key finding 1: cost as a barrier

Malawi has a predominantly agricultural economy, including a lot of subsistence agriculture, hence most rural citizens earn a living by farming [5]. Everly, a 77-year-old woman with visual and mobility impairments who was unable to farm as she once did, described challenges with regard to finding work:

“I try to go for piecework in other people’s fields; but that doesn’t work because they can’t allow me because of my disability”.

The intense physical labour involved in farming is frequently unmanageable (or at least perceived to be) for people with sensory and, especially, mobility impairments, often rendering them unemployed and unable to earn money [23].

Another interviewee, Liliana, a 21-year-old woman who had impaired mobility associated with a chronic lung condition, never worked in farming but used to run a small business. Talking about why she had to close her shop, she said,

“It has everything to do with my illness, because now I am not able to be as productive as I used to be and also we used most of the money in the business to pay for the transport to the [health care] facility”.

When income is limited, as described here, costs may become barriers. Even though the closest health care facility to most participants was a public community health centre, where services are free at the point of delivery, almost all the people interviewed described cost - be it of transport, a ‘health passport’ or pharmacy drugs - as a significant barrier to timely and adequate health care.

#### Cost of transport

A few participants, unable to walk to the health centre due to their impairment and with no outreach services to attend to their medical needs, described the need for transport in order to access health care. One man, Patrick, a 47-year-old man with severe mobility impairments following a recent stroke, revealed the financial difficulties he has since faced in trying to reach the health centre to receive care when needed:

“I travel on a bicycle which we borrow from some people in the community, and we pay for it, but sometimes it happens that we don’t have the money for hiring the bicycle, so instead I just stay here at home because there is nothing else we can do about it”.

No longer able to walk due to his stroke, Patrick could not work on the farm to earn a living anymore. Given this, the next question enquired about where he found the money to pay to travel by bicycle last time he went to the health centre:

“My sister is the one who provided the money. […] She had been asking for piecework from other people in the community, and when she worked, they paid her and she took part of the money and we used it for my transport”.

The themes of reliance on others and family support, which surface clearly here, are expanded upon later as another finding.

Another participant, Francis, was a 25-year-old man with impaired mobility and a chronic lung condition who, though able to travel to the local health centre for free, explained the financial considerations involved in reaching facilities which are further away:

“I have a bicycle which I use whenever I want to go to [the community health centre], which is closer, but one time I went to [the mission hospital], for that I sold my tobacco and I used the money for transport”.

Francis went on to explain how this hospital was often the more appropriate facility for the health care he required and highlighted the impact of his inability to pay to access it:

“It’s difficult, because it always involves money in order to travel because I have to use public transport, so money for me is an issue here, and I cannot do anything about that aspect, and that is why I resort to just going to [the community health centre] nearby for treatment”.

The close link between cost and distance is clear here and the issue of distance was frequently mentioned, especially by the participants who had a chronic lung condition. For example, Stanley, a 77-year-old man with a mobility impairment and a chronic lung condition, spoke of the difficulty associated walking to the health centre to avoid the cost of transport:

“Even to go to [the community health centre], I must sit down [to rest] several times before reaching there”

#### Cost of a health passport

In Malawi, all users of public health facilities are required to own, and take care of, a personal ‘health passport’ in which, given the absence of an established online system, their complete medical record is kept. In some discussions, the challenge of this responsibility was revealed, with Francis, 25, saying, “my mother had even lost my initial health passport because it was not constantly needed” and Stanley, 77, telling us his document was “destroyed in the rains”. For a patient to replace their lost or damaged health passport costs 200 Malawian Kwacha (the equivalent of US $0.28), which is approximately one third of the Minimum Daily Wage in Malawi [24].

Joseph, a 56-year-old man with a hearing impairment, required treatment for his symptoms of a chronic lung condition but had lost his health passport long ago. He described his need to pay for a replacement as the primary reason for not seeking health care:

“I don’t have money to go and buy a health passport to use so I didn’t go to the facility, because you are required to have a health passport in order to be able to be attended to at the facility […] The money that we find from the piecework is not even enough, so how can I take K200 from it in order to buy a health passport? We only aim at buying food”.

#### Cost of drugs

A consequence of poorly resourced public health centres - another barrier to adequate health care which will be presented as a finding in the next section - is the financial burden that frequent drug stock-outs places on patients. Users of the health care facility are required to purchase, from a private pharmacy, their prescribed medication which would have been provided for free at the health centre had it not run out of stock. Participants often drew attention to this problem and its impact in the interviews:

“The issue is the shortage of drugs, […] we are told that the drugs have run out, so we are told to buy the drugs instead. We from the villages who do not have money, […] where will we get the money from? So we end up getting back home without any assistance” (Jocelyn, a 51-year-old woman with visual and mobility impairments)

“It hurts to see that they are telling you to go and buy the drugs at the pharmacy because they don’t have the drugs, when we have gone there for such treatment […]. So when you get back home you can’t buy the drugs and you end up continuing to suffer as if you had not gone to the facility in the first place” (Everly, 77).

### Key finding 2: insufficient health care resources as a barrier

Almost all the interviews revealed that the health care provision available for this group of people was frequently inadequate to meet their medical needs, mostly due to functional, practical and systemic problems. Specifically, the main reasons for dissatisfaction with health care provision identified among our participants were unreliable drug supply, a shortage of doctors, and a lack of diagnostic testing and specialised treatment.

#### Not enough drugs

As alluded to in the comments about personal financial consequences of drug stock-outs, the health care facilities attended by our participants, and in many other parts of Malawi [25], frequently run out of drugs. The participants of this study frequently expressed frustration with the shortage of medicines:

“Here in our health facilities we have very big problems, because drugs are rarely found in the hospitals” (Ruth, a 45-year-old woman with a hearing impairment)

“There are cases where drugs run out due to the number of people who come to the facility to seek treatment” (Family member interpreter (mother) of Nathaniel, a 25-year-old man with a hearing impairment)

“The drugs are in short supply; it only takes a few days and then we hear that the drugs have run out” (Liliana, 21).

When asked how their access to health care could be improved, some participants explained that a constant supply of medication would make a considerable difference.

#### Not enough medical personnel

It became clear over the series of interviews that the inefficiency and under-staffing of the health care facilities provides a direct barrier to accessing timely health care by causing long queues and significant waiting times:

“We might wait even up to 1 o’clock in the afternoon [having arrived at 9 o’clock] before we even have the chance to meet with the doctor” (Family member interpreter (grandmother) of Esther, a 20-year-old woman with a mobility impairment)

“There is a problem because we go there when we are not feeling well and, because of the number of people there at the facility who need to be assisted […], it takes too long and for a patient it also becomes very uncomfortable” (Family member interpreter (mother) of Nathaniel, 25).

Discussions with participants revealed that there are insufficient trained medical personnel, and particularly few doctors, at the health care facilities which our participants were able to reach:

“At the moment we only have one doctor, and he has to treat over 300 patients in a day; that is a lot of work” (Everly, 77)

“There are times where those hospital attendants who sweep the facility are the ones who dispense the drugs, because the medical personnel are tied up and because there are too many people” (Family member interpreter (husband) of Ruth, 45).

Another participant, Stanley, 77, who went on to express his fear that “someone might die right at the facility” as a consequence of the extreme waiting times, highlights his doctor’s tardiness as a contributing factor:

“Another problem for us is the time the doctor reports for duty to open the clinic, most of the time he is very late to open the clinic and start providing services to people”.

The interviews also revealed that knowledge of the amount of time taken up by a visit to the health centre often deters them from attending at all.

#### Not enough diagnostic testing or specialised treatment

Being unable to regularly travel to better-equipped facilities, due to both practical and financial reasons mostly resulting from their impairments, some participants expressed disappointment at the quality and responsiveness of services accessible to them:

“I would really be very grateful if […] there was a provision for running tests in my chest so that they would be able to find out what really is my problem, and then they could tell me what is really wrong with me so that I would be given proper medication, instead of working on assumptions” (Liliana, 21)

“I would really like if there was a way of finding out what is causing all these problems that I have, and then be given proper medication for it so that my life will get back to normal” (Stanley, 77)

“The treatment that I receive is not up to my desired expectations. I really would appreciate it if they would give me an injection, because I know that injections are very effective; but I have never received any” (Samson, a 67-year-old man with impaired mobility due to arthritis).

### Key finding 3: dependence on others as a barrier

A recurring theme throughout all the interviews was that of dependence and needing support. It was clear that reliance on family, friends and the community could give rise to a social barrier to timely and adequate health care, with some participants losing their source of support, and others at the mercy of their care-giver’s availability, financial situation and, ultimately, compassion. The notion of dependence as a barrier is intrinsically linked with other barriers and, among our participants, dependence mostly presented itself as a theme in discussions about financial support, transport and distance to a health facility, and, in one case, communication.

#### Dependence on others for financial support

In response to the question ‘how do you currently earn a living?’, participants often revealed that their inability to make enough money rendered them reliant on others:

“This is a very big challenge because I am incapacitated, and I cannot do any work here at all, and now I am solely dependent on the people who are looking after me” (Patrick, 47)

“I am not able to do a lot of piecework anymore; this affects my family in terms of basic necessities; so much so that we live with little and depend on getting support from other people surrounding us here” (Joseph, 56).

Notably, discussions about the need for family or community financial support, such as these above, did not appear to be restricted to a particular age group, gender or type of impairment. One woman, Everly, 77, stressed the role that charitable donations played for her:

“There are also some well-wishers in the village who sometimes give me some money which I eventually use to buy the most day-to-day needs”.

Meanwhile, Nicholas, a 70-year-old man with a mobility impairment who lived alone and described his financial situation as “a limiting factor”, had lost his source of support:

“I was a very active young man, before my legs developed these pains and became stiff […]. Now there is nothing that I do that can really fetch me the help that I need; the one whom I depended on for financial support lives very far now”.

#### Dependence on others for transport to health care facilities

The other limiting factor that Nicholas, 70, discussed was the reduced availability and accessibility of transport since his relative stopped taking him to the health facility by bicycle. Similar accounts linking dependence on others with restricted ability to travel were given by other participants, mostly those with mobility impairments:

“[The reason why I have not gone to the health facility recently] is because of the one who takes me; he is usually not around to take me that much” (Patrick, 47)

“No I can’t [ride a bicycle anymore], but if there is someone to take me, then that can work, otherwise it will be impossible for me to do that now” (Stanley, 77).

Other interviews revealed how the need to be accompanied when walking to the health centre may also limit when one can go. The family member interpreter (grandmother) of Esther, 20, described these kinds of challenges in her explanation about how her granddaughter reaches health care:

“We walk very slowly till we reach the health facility […] because I also have a walking difficulty because my legs are no longer able to support me due to old age”

“I feel that she needs to be under constant monitoring, and that is impossible because I cannot manage to take her all the time, due to both of our mobility challenges”.

#### Dependence on others for assistance with communication

One interviewee, Ruth, 45, revealed the difficulty she sometimes faced, as someone with a hearing impairment, when trying to converse with and understand her doctor. The notion of dependence on others for effective communication with health care practitioners was presented by Ruth’s husband when he spoke as her interpreter for part of the interview:

“I write something and then give it to her to take to the doctor; so they in turn write back to me when they have not managed to communicate with her properly […]. Because she is my wife, I am able to sit her down and explain to her one by one, everything which the doctor has found out and said” (Family member interpreter (husband) of Ruth, 45).

Whilst many participants, such as Ruth, expressed gratitude towards those who supported them, some also showed signs of frustration at the lack of independence, as captured in the following quotes:

“If only this pain in my back would go; I would stop being so dependent on them and would start to do things in the way I used to do before” (Stanley, 77)

“Things have completely changed, I have become helpless because I can’t do anything by myself the way I used to do in the past” (Samson, 67).

### Attitudinal barriers

Findings about two kinds of attitudinal barriers are presented in this section: first are those on the attitudes of participants towards health care, where unfavourable health seeking behaviour may become an access barrier, and second are the findings on the attitudes of community members and health care workers towards the participants and their disabilities.

#### Unfavourable health seeking behaviour as an attitudinal barrier

When asked why they decide to seek health care, participants generally responded with variations of: “so that I would feel better” (Francis, 25).

Some participants, most of the men and one of the women, exhibited what might be considered unfavourable health seeking behaviour [26]. There were a few comments about a lack of need and a lack of motivation to seek health care as well as occasional references to the use of traditional medicine in the place of conventional health care:

“I saw that I was better off than most of them who were there [at the health care facility], so I said to myself, why am I bothering to come here for treatment when I am better than most of them? […] So I just left and got back home, and never went back” (Nicholas, 70)

“The problem started again […] and I had to go to the traditional doctor again” (Samson, 67)

[In response to: ‘what is stopping you from going to the health centre about your eyesight problems?’] “Nothing, I am just lazy when it concerns that” (Jocelyn, 51).

#### Attitudes towards disability

The participants of this study generally gave very positive responses when asked how they were treated by people in their community:

“They associate with me without any problem, and they also don’t treat me differently from everybody else, they just know to speak loudly so that I will be able understand” (Joseph, 56)

“They have accepted that I have this problem and they help me in any way they can so that I don’t have to be uncomfortable” (Liliana, 21)

“I am grateful that the people in the community accepted me following my disability, […] I could be a laughing stock […] but it is not such in this case. People here they come to chat with me, they come to see me, nobody has ever spoken badly about my illness” (Patrick, 47).

The responses given by two of the elderly male participants, were exceptions to these assurances, as they revealed feelings of social isolation within their rural communities:

“I don’t get treated the way I am supposed to; because sometimes people can say hurtful words to me because of one reason or another, and I am not very free when I live here” (Stanley, 77)

“Sometimes I just see the way that they are reacting around me, I just know that I am not needed amongst them, so I just leave and come back home and sit here by myself” (Nicholas, 70).

Furthermore, whilst participants often spoke about the shortcomings of the health care facilities, none reported experiencing any issues with the attitudes of the health care workers. The following quotes are responses to the question, ‘how are you treated by doctors and nurses when you receive care, compared to other people or compared to before you became disabled?’:

“We don’t have any problem, we are assisted properly” (Family member interpreter (grandmother) of Esther, 20)

“They treat me well […] the way [all the patients] are treated is the same” (Everly, 77)

“There is no difference; the way they do now is just the same as they used to do it before” (Jocelyn, 51).

### Time as an underlying factor

Regardless of the specific barriers each participant described, a recurring theme was ‘time’. Whether the issue was lack of it, use of it, waiting or delays, time pressures appeared to create and exacerbate health care access barriers for people with disabilities in this setting.

Some participants drew attention to the role of time within financial barriers:

“It takes a long time for the crops to reach the stage where we could be able to sell” (Liliana, 21)

“Fortunately, […] she may take several months before she goes [to the clinic] again; so we have some time to build up [money] again” (Family member interpreter (husband) of Ruth, 45).

Similarly, ‘time’ presented as a common underlying theme behind complaints about health care provision. The amount of the day a visit to the health centre requires was repeatedly highlighted as a factor contributing to dissatisfaction with health care. Time was mentioned both in reference to travelling to the facility and waiting on arrival:

“It takes me a very long time to go and come back since I walk with a lot of difficulties” (Samson, 67)

“A person may leave their home as early as possible to reach the facility in good time, but will have to wait a long time before the doctor reports for duties” (Stanley, 77)

“We usually wait a longer time since there are many people who go there since it is a public health facility” (Ruth, 45).

Some participants described how the long waiting times mean patients’ conditions often decline considerably before they receive any medical attention. Samson, 67, gave a different, practical, reason for time being an important consideration:

“We want to be there early so that we become one of the first people to be attended to, so that I […] beat the scorching sun on my way back”.

Finally, participants who discussed the dependency that results from their disability often explained it is how the lack of time or availability of the person who assists them that causes reliance on others to be an access barrier:

“Whenever I ask him to take me, he only postpones to the other day, and in that way, days go past” (Patrick, 47) […] “We are waiting for the cousin who takes him to find time so that he can take him back [to the health care facility]” (Family member interpreter (sister) of Patrick).

## Discussion

The findings of the study show that our participants face multiple, complex barriers to accessing healthcare. The three key barriers identified – cost, insufficient health care resources, and dependence on others – are discussed and interpreted with reference to Thaddeus and Maine’s Three Delays and Levesque et al.’s accessibility model. The discussion on the impact of each of the key barriers on our participants’ pathway to care is followed by a visual summary of the correlations between the barriers and the two models. Following this, we explore the role of the attitudinal barriers, the identification of time as an underlying factor, and the possibility of interactions between the barriers. Finally, we discuss the limitations of the study and the implications of the findings.

### Discussion of cost as a barrier

Consistent with what has been repeatedly shown in other studies in rural Malawi and similar contexts [10, 27, 28], the participants’ quotes highlight the significance of financial barriers to health care access for people with disabilities living in such settings, with Levesque et al.’s ‘ability to pay’ [9] proving an aspect of accessibility that is not only relevant for people using private services but also for those who attend public facilities. This finding complements the results of a recent study on the accessibility of health services for people with disabilities in Malawi [28], which found that cost was the “one of the most important barriers”.

The accounts given by Francis and Patrick reveal that cost has the potential not only to provide a barrier to the receipt of timely health care also to hinder access to appropriate and adequate care. The financial barriers associated with distance to a suitable medical centre provide a clear example of how travel costs may ‘delay arrival at the health facility’ [8], the second of Thaddeus and Maine’s three delays, as well as how they hamper the ‘ability to reach’ and ‘ability to pay’ [9] aspects of accessibility, as described by Levesque et al..

Whilst many participants made reference to the issue of the distance to a health care facility and the cost required to cover it, when asked what they thought would improve the accessibility of health care, none responded with ideas about having a closer facility available. A systematic review by Bright et al. found that introducing services at or close to home was an effective intervention for improving children’s access to health care in low- and middle-income countries [29], and it is reasonable to expect that this would also be the case for people with disabilities. Therefore, the fact that our participants did not make this suggestion may possibly reflect assumptions that an increase and improvement in the number and distribution of facilities are improbable prospects.

Given that all of the elderly participants (those aged >65 years) in this study emphasised the difficulties posed by the journey to the facility, it is important to consider the role that age may play in determining the extent to which distance from a health care facility acts as an access barrier. Munthali et al. [25] find that old age is an independent barrier to health care access and describe how the long distances to facilities and poor transport options have a negative impact on the health seeking behaviour of elderly people, especially those with age-related impairments, like some of the participants of this study.

The complaints about the cost of a health passport highlight how poverty, which may be both a consequence and cause of disability [30], alongside system-level administrative barriers, can prevent access to even the most basic public health centre in this setting.

Although the two quoted descriptions of how the cost of drugs acts as a barrier are both from women with visual and mobility impairments, similar experiences were alluded to by participants of all other profiles, indicating that the challenge of the ‘affordability’ dimension of accessibility cuts across gender, age, type of impairment and lung health status within this small sample.

Importantly, financial barriers are also likely to affect many people in this setting who do not have a disability, and the extent to which this might be the case has not been explored in this study since personal accounts from people without disabilities were not collected. It is therefore difficult to establish how the weight of cost as a barrier to health care access may compare for the two groups. Nevertheless, the particularly significant impact that health care costs have on people with disabilities is well-recognised, for not only are these people more likely to be poor but they are also likely to face higher costs due to greater additional needs for access (such as transport and carers) and specialised treatment, and increased frequency of use (as a result, both directly and indirectly, of the disability) [28, 31, 32].

### Discussion of insufficient health care resources as a barrier

The recurring pattern of remarks about the need for an improvement in drug supply emphasises the significance of the third of Thaddeus and Maine’s Three Delays, ‘delay [in] the provision of adequate care’ [8], within participants’ pathways to health care. This delay appears to be exacerbated by the fact that the insufficiency in medical personnel causes long waits at the facilities. Not only is it possible for patients’ conditions to deteriorate over the period of time they are waiting, as some participants described, but it also may take up to a whole day away from potential work for themselves and whoever might be accompanying them, which could signify a close link with financial barriers.

In addition to inefficient health care delivery increasing the ‘delay [in] the provision of adequate care’ [8], the interviews revealed that knowledge of the amount of time taken up by a visit to the health centre negatively influences participants’ health seeking behaviour. This observation, which more often appeared to be the case among men than women, points towards a greater ‘delay [in] the decision to seek care’ [8]. In this way, the serious mismatch between supply and demand has been found to provide both a direct and indirect barrier to accessing timely health care for our participants.

The quotes in which participants describe a lack of diagnostic tests and specialised treatment highlight a further extension of the ‘delay [in] the provision of adequate care’ [8] and portray ‘appropriateness’ [9] as a limiting factor of accessibility, stressing how even if and when health care facilities are reached, receipt of ‘proper’ health care may not be guaranteed. It is important to acknowledge, however, that the dissatisfaction with health care described by participants may result not only from the underperformance of health care facilities but also, sometimes, from unrealistic patient expectations.

In a study by Saleh et al. [33], high expectations about the availability of diagnostic tests and curative treatment are reported to influence patients’ perceptions of health care service quality in Malawi, with patients especially attracted to the idea of receiving treatment in the form of an injection. In the current study, some participants alluded to a presumption that health care providers could cure their condition and hence, they expressed frustration about the fact that this had not been achieved. Saleh et al. find that these expectations are particularly held by users of private health care facilities [33], but insufficient data on the usage of private facilities by our participants meant that this trend was not tested here.

The findings show that insufficiencies in health care provision, which may be viewed as flaws in the ‘availability and accommodation’ and ‘appropriateness’ dimensions of accessibility [9], provide a major barrier to the attainment of timely and adequate health care for participants of this study. It is important to note however, that the issues the participants described affect all users of health care services, rather than only those with disabilities. Nevertheless, it is probable that such barriers are experienced more frequently by people with disabilities due to their statistically increased need for health care [2], together with a likely reduced ability to afford drugs elsewhere, to pay for private and better-staffed health care and to access distant and better-equipped facilities, as a result of their disability.

Meanwhile, it is possible that the reported positive attitudes of Malawian health care workers towards disability can be partially attributed to recent campaigns to improve their attentiveness towards the needs of people with disabilities, as described by Grut et al. [11].

### Discussion of dependence on others as a barrier

Although caregiver dependence is recognised as a significant aspect of the lived experiences of many people with disabilities in similar settings to our participants [34, 35], there is currently limited emphasis in the surrounding literature on its impact on health care accessibility. It is clear from this study, however, that dependency on others frequently exposed our participants to the inconsistency and instability that often accompany charity, demonstrating how the need to depend on others’ generosity may act as a barrier.

Some interviews revealed how participants may have to ‘delay the decision to seek care’ and ‘delay arrival at the health facility’ [8] if their disability means that they must be accompanied to the health centre, for they are dependent on the availability of somebody else. Moreover, the quote given by the grandmother of Esther draws attention to the way in which the health status of the supporter may also be a factor contributing to the ability of the dependant to access health care.

The report given by Ruth’s husband on his wife’s difficulty communicating alone with health care practitioners showed how dependence on others may ‘delay the provision of adequate care’ [8]. It is clear that without reliable support, communication difficulties may provide a significant barrier to the successful receipt of adequate health care.

Highlighted by the participants’ accounts is the way in which reliance on other people may contribute to all three of Thaddeus and Maine's ‘delays’ [8] and may impact on the patient’s abilities to ‘seek’, ‘reach’ and ‘engage [in]’ health care [9]. Nevertheless, in discussing how dependency may hinder health care accessibility, it is also essential to acknowledge the ways in which support from others acts as a facilitator for health care access among people with disabilities, and certain accounts drew attention to the enabling benefits of appropriate support, when available, especially that provided by family members.

In our study, the main expressions of frustration about dependency due to disability were given by men, in particular those who had developed their impairment later in life and had since experienced a change in their ability to exercise their fundamental right to health [7, 28]. Recognition of the adjustment challenges and mental health difficulties which may be experienced by many such people with disabilities further stresses the necessity of equitable health care accessibility [36].

### Summary of correlations between the key findings and the accessibility models in the literature

A summary of the correlations identified between the barriers found in this study and two of the health care accessibility models described in the literature is provided in Table 3.

**Table 3:** Correlations with Thaddeus and Maine’s and Levesque’s models

Barrier to health care access (Key Findings of this study)	Delays (as described in Thaddeus & Maine’s Three Delays model [8]) extended by this barrier	Dimensions and abilities of accessibility (as described by Levesque et al. [9]) hindered by this barrier
Key Finding 1: Cost as a barrier	2nd delay (arrival at a health facility)	Affordability Ability to reach Ability to pay
Key Finding 2: Insufficient health care resources as a barrier	1st delay (the decision to seek care) 3rd delay (the provision of adequate care)	Availability and AccommodationAppropriateness
Key Finding 3: Dependence on others as a barrier	1st delay (the decision to seek care) 2nd delay (arrival at a health facility) 3rd delay (the provision of adequate care)	Ability to reach Ability to pay Ability to engage

A table showing the correlations between the barriers identified in this study, Thaddeus and Maine’s ‘delays’ and Levesque et al.’s ‘dimensions and abilities’

### Discussion of the attitudinal barriers

Regarding the attitudes of people with disabilities towards seeking health care, the accounts given by some of the participants illustrate how social and cultural factors may contribute to unfavourable health seeking behaviour, a finding supported by similar studies [26, 28]. It is also possible that the perceived barriers to appropriate health care access, including understandings of the quality of care provision, can become manifest in the health seeking behaviour of people with disabilities in this setting [8]. Notably, however, this may also be the case for people without disabilities, as found by Sanou et al. in Burkina Faso, a low-income sub-Saharan African country which, like Malawi, has a significantly under-performing health system [37, 38].

With regard to the perceived attitudes of community members towards disability, the findings of this study contrast with those of other studies conducted in Malawi [35, 39], in that our participants generally reported that their community was supportive, and that even those who did experience social exclusion because of their disability did not find it affected their access to health care. Some studies have found that stigmatisation and discrimination at the community level discourages people with disabilties from attending health services [40, 41]. It might be inferred, therefore, that the feelings of exclusion and restricted freedom described by some of our participants could ‘delay the decision the seek care’ but, when asked, our participants denied that this was the case. As such, attitudes towards disability are not found to provide a barrier to health care access in this study.

Finally, regarding attitudes of health care workers towards people with disabilities, the participants of this study denied any issues and although many expressed frustration about the limited resources at health care centres, no complaints were made about the way in which they, as people with disabilities, are treated by staff. This is in contrast with much of the literature [2, 6, 10], but in agreement with findings by Grut et al. [11]. It is also in keeping with the positive reports which most of the participants in this study gave about community attitudes towards disability.

### Discussion of time as a common underlying factor

The repeated references to the challenge of time in all of the interviews rendered it necessary to include as an underlying factor contributing to the barriers. The role of time in the participants’ experiences, however, is complex, and lack of time is a frustrating problem to identify because it is so difficult to address. Time often represents money, hence it was interesting to note that when the problems associated with the amount of time taken to visit the health centre were described, loss of potential working hours was not explicitly mentioned by participants. It is likely that this is because some participants were unable to work at all but also because there were other, more direct consequences. Some, for example, described how the long waiting times mean patients’ conditions often decline considerably before they receive any medical attention. Nevertheless, the theme of time, which is central in Thaddeus and Maine’s framework in the form of delays [8], remains a crucial factor influencing barriers to health care access.

### Interactions between barriers

The relationships between different barriers to accessing timely and adequate health care identified in this study are complex and dynamic. Financial barriers, for example, may be exacerbated by the distance to the health care facility and may link closely with the barrier of insufficient health care resources, both because cost may influence the choice of facility and because it might prevent the person from accessing essential drugs or tests. Furthermore, financial difficulties may influence the degree to which a person with a disability depends on others which, in turn, may affect their health-seeking behaviour and restrict their ability to travel to appropriate health care facilities in a timely manner.

Whilst, given the size and nature of this study, we must be cautious not to declare the existence of associative or causal relationships between the findings, it is clear that the barriers cannot be considered in isolation and, subsequently, strategies to address them demand combined implementation.

### Limitations

The limitations of the study include the use of family member interpreters, the exclusion of people with documented cognitive impairments or mental illnesses, and the relatively small geographical area in which the 12 participants lived, meaning that they mostly attended the same health care facilities. It is important to understand that this last point limits the generalisability of the findings, however the fact that this study mostly supports the conclusions of the current surrounding literature from similar settings elsewhere in Malawi [10, 11, 28], suggests that the key barriers it identifies are likely to play important roles in health care accessibility for people with disabilities in other rural parts of the country as well.

In particular, we acknowledge that the inclusion of interviews conducted via family members means that the findings cannot be interpreted in quite the same way as if all the interviews had been directly from the people with disabilities, with the help of a sign language interpreter, for example. It is inevitable that the responses given by family member interpreters reflect that person’s own perspective and experiences on health care accessibility to some extent [42]. We reason, however, that the comments made by close relatives carry their own unique value and we feel confident that the responses given are closely representative of the disabled participants’ own opinions based on our observations of the verbal and non-verbal communication that the family members and the people with disabilities shared between questions throughout the interviews. Nevertheless, with this limitation in mind, care was taken to ensure that all the reported findings of the study were based primarily on direct quotes from people with disabilities and none were solely supported by quotes from family member interpreters.

A further limitation is the fact that the study did not include any interviews with health care workers which might have provided a useful alternative perspective. Moreover, by not interviewing people without disabilities (other than family members acting as interpreters), we are unable to accurately explore the extent to which our findings are specific to people with disabilities.

Finally, although only 12 people were recruited for interview, the sample size may be considered a strength of this study for it was small enough to allow in-depth data collection and analysis yet large enough for there to be considerable variation in participant profiles.

### Implications

The implications of the findings include a requirement for better implementation of social security systems in Malawi to reduce the financial barriers to health care so commonly experienced by people with disabilities, frequently resulting from limited work opportunities. In addition, the study highlights a pressing need for improved drug supply to health care facilities, especially in rural areas, as well as the scaling-up of accessible services and improvement in the number and distribution of trained medical personnel – measures which would likely be of benefit to people without disabilities, as well as those with disabilities. Finally, it is possible that outreach schemes and community engagement in a ‘buddying’ system involving volunteers and health care workers may overcome some of the precariousness and vulnerability associated with dependence on others and so improve health care access for people with disabilities.

Importantly, given the co-existence of different barriers described in each interview, and the close interconnections between accessibility challenges, the proposed strategies may have limited effectiveness in isolation. Instead, we recommend a multi-dimensional approach which facilitates integrated employment of interventions. In this way, striving for equity in health care accessibility, Malawi may continue to work to reduce health inequalities.

## Conclusions

Barriers to accessing timely and adequate health care in rural Malawi may be encountered at various points along a pathway to care and our findings suggest that people with mobility impairments, sensory impairments, or both, face many of these barriers. The three main barriers identified in this study are: 1) cost of a visit to a health care facility, 2) insufficient health care resources, and 3) dependence on others. Underlying these, and the attitudinal barrier of unfavourable health-seeking behaviour, there is a general theme of time limitation as a contributing factor.

Most of the different types of access barrier were experienced by participants of diverse profiles, suggesting that the challenges were significant enough to override differences in gender, age or type of impairment within this cohort. For people with a chronic lung condition alongside a mobility or sensory impairment, the main barriers were the same as for the other participants. Whilst it is recognised that financial barriers (especially the cost of drugs) and experiences of inadequate health care (doctor shortages in particular) are likely to apply to the population at large, we maintain that for people with disabilities the challenges may often have a “greater negative impact” [11]. Dependence on family, friends and the community is presented as a central yet frequently under-acknowledged barrier to health care access which is especially pertinent for people with disabilities. With the social model of disability definitions in mind [14], our study shows that how the prominent access barriers faced by our participants cause their impairments to become disabilities.

The execution of this study has met a crucial need for more in-depth exploration of the health care access barriers faced by people with mobility, sensory, or both, impairments in rural low-income settings. The study’s findings shed light on the significance and relevance of access barriers identified in the surrounding literature for people with these impairments in Dowa district, central Malawi. Whilst there is a need for further research to explore the efficacy of the proposed interventions, we hope that the findings of this study have the potential to inform local policy by indicating areas for improvement in order to reduce some of health inequalities people with disabilities in rural Malawi currently face.

## Supplementary information

**Additional file 1.** Topic Guide.

## Data Availability

Due to the level of detail in the qualitative transcripts from this study, even with direct personal identifiers removed, the stories and descriptions of participants within the transcripts makes them potentially identifiable, as well as containing sensitive and confidential patient information. We do not have ethical approval for the sharing of raw data sets from our institutional ethics committee and we have therefore deemed these raw data unsuitable for sharing publicly. We have tried to share as much data as possible through quotes within the text. The dataset is available on reasonable request from Julie Irving (Julie.irving@lstmed.ac.uk) who is an institutional representative and will hold a copy of the data and respond to external access requests. To ensure long-term data storage and availability it will be held by two of the authors, Josephine Harrison and Rachael Thomson, as well as Ms. Julie Irving.

## Abbreviations

REACH: Research for Equity And Community Health
WGSS: Washington Group Short Set
WHO: World Health Organisation
AAAQ: Accessibility, Availability, Acceptability and Quality
PAL: Practical Approach to Lung health intervention
PAL+: Practical Approach to Lung health intervention plus community outreach intervention
GPS: Global Positioning System
COMREC: College Of Medicine Research and Ethics Committee
LSTM: Liverpool School of Tropical Medicine
NGO: Non-Governmental Organisation
