# Supplementary material for: Access to health care for people with disabilities in rural Malawi: what are the barriers?
Source: BMC Public Health. 2020 Jun 1;20:833. doi: 10.1186/s12889-020-08691-9 (PMC7268500; doi:10.1186/s12889-020-08691-9)
Supplement: Supplementary file 1 — Additional file 1. Topic Guide. [file 12889_2020_8691_MOESM1_ESM.docx]

**Additional File 1. Topic Guide.**

Contents

Topic guide (English version) ………………………………………………………………………………………… 2

Topic guide (Chichewa version) ……………………………………………………………………………………. 6

**Topic guide (English version)**

**Project Background**

**Title**

Access to health care for people with disabilities in rural Malawi: what are the barriers?

**Aim**

To explore the barriers to timely and adequate health care experienced by people with mobility or sensory impairments, or both, in rural Malawi

**Objectives**

1) To make comparative observations about the barriers reported by participants of different gender, age and type of impairment (mobility, sensory, or both), as well as between those who have a chronic lung condition and those who do not.

2) To explore how the barriers may interact with each other and investigate the possibility of a common underlying factor.

**Interview Details**

Interview ID number:
Date of interview:
Household number:
Village number:
Interviewer:
Interpreter:

**In-depth interview**

This topic guide is split into 3 sections. If the participant’s response to the question below reveals that they have had their impairment for as long as they can remember, just use sections 2 and 3. If, however, there has a been a period of their life during which they did not have the impairment, use all 3 sections.

- When did you start finding it difficult to walk/move/see/hear?

**1) Life before development of mobility or sensory impairment**

Health**:**

- How was your health before you developed the disability?
- PROMPTS: Childhood; Any other health conditions; Comparison with peers (perceived relative health); Presence or absence of a chronic lung condition

Livelihood:

- How did you make a living?
- PROMPTS: Schooling; Family; Employment; Single occupation or multiple occupations; Reliability of income source

Day-to-day activities:

- Describe your day-to-day life prior to your development of this impairment.
- Which household jobs did you carry out?
- How did you feel in general?
- PROMPTS: Physically strong/weak; Emotionally strong/weak; Confident/relaxed/anxious

Transport:

- How did you generally get around? / What were your main modes of transport?
- PROMPTS: Walking/cycling; Public transport; Independent/Dependant on family member?

Family life:

- What was your role within the family?
- PROMPTS: Feelings of being relied upon/important/respected; Supporter or dependant

Community life:

- What role did you have in your community before you developed your impairment?
- How were you treated by other members of the community?
- PROMPTS: Associations with certain groups; Positions of responsibility; Social interactions

Engagement with health services:

- When you are feeling unwell, what do you normally do?
- Of the times that you needed to, how often did you actually go to the doctor?
- PROMPTS: Every time/sometimes/never; Reasons why

**2) Life following the development of a mobility or sensory impairment**

Health:

- How did the development of the impairment make you feel?
- How has the amount of health care you need changed?

Livelihood:

- How has your disability affected your occupation, employment or livelihood activities?
  - PROMPTS: Changes in capability and performance; Feeling of being respected/valued; Provision of support; Discrimination

Day-to-day activities:

- Describe how your day-to-day life has changed following the development of your impairment.
- Are there any changes regarding which household jobs you carry out?
- How much of your day do they take up compared to before?

Transport:

- How does your disability affect your movements and travel? / What is your main mode of transport at present?
- PROMPTS: Easy/difficult; Use of walking aids; Degree of stamina; Public transport; Independent or reliant on assistance/accompaniment by family member

Family life:

- How does your disability affect your role within the family?
- PROMPTS: Feelings of importance; Respect; How family members treated you compared to before

Community life:

- How your role within the community been affected, if at all, by your development of an impairment?
- How does your disability affect how you are treated by other members of the community? / How do people in your community treat you now compared to before?
- PROMPTS: Acceptance or discrimination; Respect or ridicule; Inclusion or isolation
- What do people in the community understand by the term ‘disability’? / How do people in this community generally behave towards disabled people?

Engagement with health services:

- Following the development of your disability, which health care facility do you choose to visit if you need to see a doctor?
- If your choice of health care facility has changed compared to before you became disabled, can you tell me why?
- Of the times that you need to, how often do you actually see a doctor?
- PROMPTS: Every time/sometimes/never – why?

**3) Life with both a mobility or sensory impairment: Impact on access to health care**

Frequency of requirement for health care:

- How often do you require medication or treatment? / How often do you need to see a health care practitioner?
- When did you last see a health care practitioner and what did the visit involve?

Decision to seek health care:

- Who in the family makes the decisions about going to the health care facility?
- How unwell do you usually feel when you decide you must to see a doctor?
- What influences your decision to go to the health care facility
- PROMPTS: Role within the family and community; Employment; Performance

Reaching a health care facility:

- How do you travel to and move around the health care facility?
- Who (if anyone) travels with you when you go to the health care facility?
- How much of your day does a round trip to the health care facility take up?
- How much does a visit to the health care facility cost? / Where do you find the money to pay for a visit to the health care facility?

Receiving adequate health care:

- Once you have reached the health care facility, how long does it take for you to receive the treatment or care you need?
- How are you treated by the medical personnel at the health care facility?
- PROMPTS: Same or different to others; Same or different to before; Respectful or dismissive
- How, if at all, has this changed compared to before you developed your disability?

Ways to improve

- How do you think your access to health care could be made easier? / What changes could the government make to improve your ability to access health care?

**Topic guide (Chichewa version)**

**1) Life before development of physical disability and chronic lung condition**

- Ndiliti limene munayamba kuvutika ndi kuwon/kuyena/kumva ?
- Kodi pakhomo pano kweni kweni mumadilrauguochiyani kuti mupeze ndalama.
- Musanayambe kukhala ndi mauuto oterewa, moyo wanu watsiku ndi tsiku umakhala bwanji ?
- Ndiye mumagwiritsa ntchito chani nkhani ya mayendedwe ?
- Udindo wanu ndiwotani pabanja pano ?
- Nanga udindo wanu unali wotani mmundzi muno musaakhal ndi uuto limene muli nalo panoli
  Or, Mmatenga mbali yotani mmudzi muno
- Musanakhale ndi uutoli, mukafuna kupita kuchipatala, kayendedwe kanu kamkhala Kotani kuti mukaepeze thandizo

**2) Life after the development either a physical disability or chronic lung condition**

- Uuto lanu limene muli naloli, lakhudza bwanji moyo wanu watsiku ndi tsiku makamaka mukapezedwe kachuma ?
- Tafotokozani mmene moyo wanu wasinthira potsatira vuto limene inu mwakumana nalo panopa.
- Uuto muli naloli, lasinthitsa motani kayendedwe kanu pakhomo pano, komanso pamene mukupita malo osinyanasiyana ?
- Uuto lanuli lasintha bwanji udindo wanu umene mumakhala awo pabanja pano ?
- Kodi anthu mdera lino amakhala nanu bwanji panopa mmene munakhala ndi uuto limeneli ?
- Kodi anthu amdera linolo amadziwapo chani za ulumali komanso uuto la matenda a mchifuwa ?
- Ndiliti lomwe munawuzidwa kuchipatala za vuto lomwe muli naloli ?
- Kutsatira zomwe munauzidwazo, mumapita kangati kuchipatala kti mukapeze chithandizo ?

**3) Life with both a physical disability and chronic lung condition: Impact on access to health care**

- Ulendo wanu omaliza kukapeza thandizo ku chipatala unali liti? Ndipo mutafika kumeneko munathandizidwa motani ?
- Chimene chimakupangitsani kweni kweni kuti muzipta ku chipatala kukapeza chithandizo pa uuto lomwe muli naloli ndi chiyani ?
- Mumayend bwanji papita ku chipatalako ?
- Mumapita ndi ndani ?
- Mumagwiritsa ndalama zochuluka bwanji pa ulendo wanu wonse wa ku chipatala ?
- Mukafika ku chipatala, mumadikira nthawi yayitah bwanji musana wonane ndi adotolo ?
- Adotolo kapena a nurse amakulandirani motani mukapita ku chipatala ?
- Kodi mmen amakulandilirani panopa, ndli kusiyanitsa kulikonse ndi mmene amakulandilirani poyamba musanakumane ndi uutoli ?
- Mkuwona kwanu, mukuganiza kuti pakuyenera kuchitika chani kuti kapezedwe ka thandizo la chipatala kakhale kosauuta ?
